# Systemic trade-offs between core and accessory genomes govern stress adaptation in *Rhodococcus erythropolis*

**DOI:** 10.1128/msystems.00137-26

**Published:** 2026-06-09

**Authors:** Xiaoran Cheng, Huan Liu, Xu Qiu, Wanjing Wu, Haiyang Hu, Ping Xu, Hongzhi Tang

**Affiliations:** 1 State Key Laboratory of Microbial Metabolism, School of Life Sciences and Biotechnology, Shanghai Jiao Tong University 553742 , Shanghai, People's Republic of China; Institute of Urban Environment, Chinese Academy of Sciences, Xiamen, China; Institute of Microbiology, Chinese Academy of Sciences, Beijing, China; Nanjing Agricultural University, Nanjing, Jiangsu, China

**Keywords:** pangenome, growth-defense trade-off, *Rhodococcus erythropolis*, systems biology

## Abstract

The genus *Rhodococcus* is a premier biotechnological chassis for organic pollutant bioremediation and natural product biosynthesis, yet the systemic genetic basis of its stress resilience remains poorly defined. Here, we elucidate adaptive strategies in *Rhodococcus erythropolis* by integrating pangenomics with multi-omics and phenotypic analyses. We refined *R. erythropolis* taxonomy using average nucleotide identity across 671 genomes and constructed a high-quality pangenome that exhibits an open architecture, with continuous expansion of the accessory cloud genome via horizontal gene transfer to enable environmental adaptation. Using *R. erythropolis* strain XP as a representative model, we confirmed broad physiological robustness, including tolerance to multiple heavy metals [Ni(II), Zn(II), Pb(II), Cu(II), and Cr(VI); minimum inhibitory concentrations, 2–7 mM], wide pH ranges (5–11), and high salinity (1.5 M NaCl). Integration of comparative transcriptomics with weighted gene co-expression network analysis revealed the transcriptional basis of this resilience. A key growth-regulatory module (ME1), dominated by evolutionarily conserved core genes (68.8%), including essential cell division components, was identified. Under severe stress, this core module is strongly downregulated, coinciding with stress-induced filamentation. These results expose a fundamental evolutionary trade-off: repression of vertical propagation via core functions enables preferential deployment of accessory cloud genes that confer resistance. Collectively, this study links pangenome plasticity to physiological trade-offs and provides a conceptual framework for optimizing *R. erythropolis* in industrial applications.

**IMPORTANCE:**

Microorganisms must continually balance rapid growth with survival under stress, yet the genomic architecture underlying this trade-off remains unclear. By analyzing 671 genomes to refine the taxonomy of the biotechnologically important bacterium *Rhodococcus erythropolis* and integrating multi-omics data, we demonstrate that this physiological balance is mirrored by an evolutionary division of labor. The conserved core genome predominantly governs growth, whereas the horizontally acquired accessory cloud genome drives stress resistance. Under severe stress, the bacterium downregulates core cell division machinery to prioritize resources for activating its accessory defense repertoire. This work establishes a direct link between pangenome evolution and cellular fitness, offering theoretical guidance for engineering robust microbial chassis.

## INTRODUCTION

*Rhodococcus* is a gram-positive, aerobic, non-spore-forming actinobacterium with high G + C content (61%–70%) (1, 2) that inhabits diverse environments, including soil, water, and host-associated niches (3–6). Their ecological success stems from exceptional metabolic versatility and environmental robustness (7), traits that have established *Rhodococcus* as a leading biotechnological chassis for bioremediation and the biosynthesis of high-value compounds (8). Large genomes encode extensive enzymatic repertoires capable of degrading complex xenobiotics such as petroleum hydrocarbons (9), aromatic compounds (10), and lignocellulosic substrates (11). Current research largely emphasizes enzyme optimization (12) and metabolic engineering (13); however, the genetic architecture and regulatory logic that underpin this adaptability remain incompletely understood.

Pangenomic analysis provides a systematic framework to address this gap by capturing the complete gene repertoire of a species and resolving patterns of conservation and diversity (14, 15). Pangenomes are typically partitioned into a conserved core genome, useful for phylogeny and species delineation, and an accessory genome that often encodes niche-specific functions, including environmental adaptation and resistance (16). This approach has revealed core–accessory division of labor in diverse bacteria. For instance, the accessory genome of *Escherichia coli* and *Acinetobacter johnsonii* drives environmental adaptation and habitat-specific traits (17, 18), and variable genome elements of pathogens such as *Mycobacterium tuberculosis* and methicillin-resistant *Staphylococcus aureus* underpin virulence and drug resistance (19, 20). Despite these advances, how pangenome architecture translates into physiological stress responses, particularly the regulatory logic governing resource allocation between growth and defense, remains underexplored.

Within this genus, *Rhodococcus erythropolis* offers an ideal system to bridge this gap. It is a metabolically versatile species with broad environmental adaptability, underpinned by an enzymatic repertoire that enables diverse bioconversions (7, 21, 22). *R. erythropolis* strain XP has previously been identified as a strong candidate for biodesulfurization (23) and alkane degradation (24). Although its metabolic capabilities are well characterized, the genetic determinants of its environmental resilience are not. Moreover, the taxonomy of *R. erythropolis* and closely related taxa has long been ambiguous, as 16S rRNA sequencing lacks resolution among near neighbors (25, 26). Robust genome-based classification using metrics such as average nucleotide identity (ANI) is therefore essential to connect genomic predictions with *in vivo* stress physiology (27).

Here, we applied an integrated multi-omics strategy to dissect stress adaptation in strain XP. We first performed phylogenomic analyses to refine *R. erythropolis* taxonomic boundaries and constructed a curated pangenome revealing an open, horizontally driven genetic architecture. We then experimentally validated the strain’s resistance to heavy metals and environmental extremes. Finally, comparative transcriptomics coupled with weighted gene co-expression network analysis (WGCNA) uncovered the regulatory logic underlying survival. Our results identify a core growth module (ME1) whose stress-induced repression enables resource reallocation toward accessory, often plasmid-mediated, resistance functions, revealing an evolutionary trade-off between growth and stress tolerance.

## RESULTS

### Redefining *R. erythropolis* taxonomy through large-scale genomics

**Fig 1:**
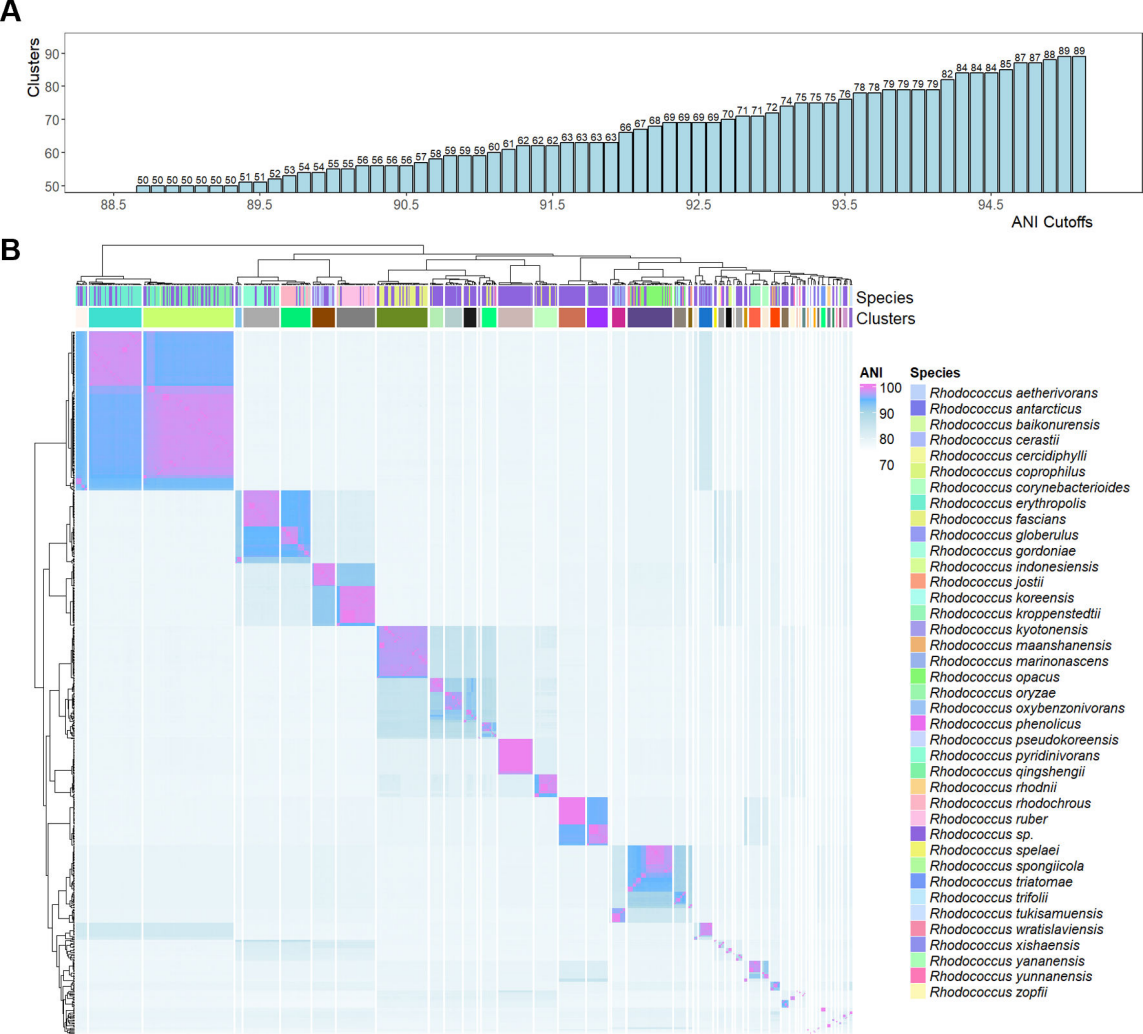
Taxonomic clustering of 671 *Rhodococcus* genomes based on pairwise average nucleotide identity (ANI). (**A**) Number of ANI clusters across cutoff values ranging from 88.7% to 95%. (**B**) Reclassification of 671 genomes into 50 clusters at an 89.3% ANI threshold, compared with NCBI species annotations.

To establish a robust genomic framework, we analyzed 671 *Rhodococcus* genomes from NCBI, excluding atypical assemblies and metagenome-assembled genomes (MAGs). Systematic evaluation of ANI thresholds (85%–97%, 0.1% increments) revealed that the exceptional genomic diversity of the genus precludes a single universal cutoff. Instead, the number of inferred taxa plateaued at 50 as ANI values decreased to 89.3% and remained stable at 88.7% (Fig. 1A). Clustering heatmaps of the 50 most abundant taxa showed strong concordance between these ANI-defined clusters and current classification schemes while also providing a rigorous basis for delineating novel groups. Notably, many newly resolved taxa originated from previously unclassified genomes, indicating substantial hidden diversity within the genus *Rhodococcus* (Fig. 1B).

Within this framework, *R. erythropolis* and *Rhodococcus qingshengii* exhibited particularly high genomic similarity, a feature that has long obscured their taxonomic boundaries. Our analysis resolved the 99 genomes annotated as *R. erythropolis* in public databases into three distinct lineages: (i) authentic *R. erythropolis sensu stricto* (38 genomes), (ii) a major clade reclassified as *R. qingshengii*, and (iii) a previously unrecognized phylogroup. To ensure taxonomic accuracy, 61 misclassified genomes were excluded. ANI-based clustering identified 52 genomes as *R. erythropolis sensu stricto*; of these, 38 were already annotated as *R. erythropolis* in NCBI, whereas 14 were originally annotated as *Rhodococcus* sp. but clustered with *R. erythropolis*. This curated data set of 52 high-confidence genomes served as the foundation for subsequent pangenome analyses.

### Open pangenome architecture enables horizontal gene transfer-mediated adaptation

**Fig 2:**
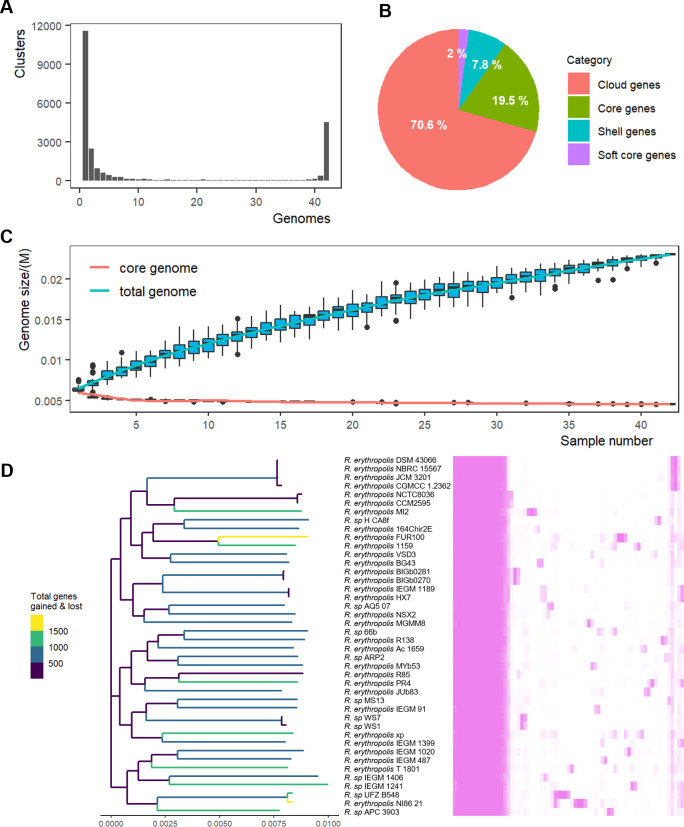
Pangenome dynamics of *R. erythropolis*. (**A**) Distribution of 23,081 gene clusters across the genomes, showing 11,571 strain-specific clusters and 4,507 core clusters shared by all genomes. (**B**) Proportions of core (*n* ≥ 99%), soft-core (99% > *n* ≥ 95%), shell (95% > *n* ≥ 15%), and cloud (*n* < 15%) gene clusters. (**C**) Gene accumulation curves for the pangenome (blue) and core genome (red) with sequential genome additions. (**D**) (Left) Phylogenetic tree of *R. erythropolis* with branches colored by inferred gene gain and loss events using maximum parsimony; (right) gene presence–absence matrix across genomes (pink, present; white, absent).

To ensure analytical robustness, the 52 verified genomes were further filtered by completeness, resulting in 43 high-quality genomes for final pangenome construction (Fig. S1). This analysis identified 23,081 gene families (Fig. 2A), revealing a pangenome overwhelmingly dominated by the accessory cloud genome, which accounts for 70.6% (16,302 families) of the total diversity. In contrast, the evolutionarily conserved core genome comprises only 19.5% (4,507 families) (Fig. 2B). The pangenome accumulation curve conforms to Heaps’ law with an *α* value of 0.36 ± 0.04 (Fig. 2C), unequivocally classifying *R. erythropolis* as possessing an open pangenome.

To elucidate the forces driving this expansion, we inferred gene gain and loss events along the phylogeny using Panstripe (28). Core branch lengths were strongly correlated with gene exchange events (*P* = 7e^−6^) (Fig. 2D), providing statistical evidence that horizontal gene transfer (HGT), rather than vertical inheritance alone, is a dominant driver of genome evolution in this species. Complementary analysis with HGTector (29) identified 1,130 HGT-derived gene clusters, of which 66.3% originated from the class Actinomycetes. Importantly, most of these genes (749, ~66%) reside within the variable accessory genome (cloud, shell, and soft-core), whereas only 381 are part of the core genome. This skewed distribution indicates that HGT primarily expands the adaptive genetic repertoire while leaving the conserved cellular machinery largely intact.

Functional enrichment analyses (Gene Ontology [GO] and KEGG) further clarified the adaptive role of the cloud genome (Fig. S2). Core genes were predominantly associated with essential housekeeping functions, whereas the cloud genome was significantly enriched for pathways linked to environmental adaptation. GO terms were dominated by processes involving transmembrane transport of inorganic ions and anions (Fig. S2a), while KEGG pathways were enriched for the biodegradation of complex xenobiotics, including fatty acids, toluene, and chloroalkanes (Fig. S2b). This functional compartmentalization supports the view that the cloud genome acts as a flexible reservoir enabling metabolic breadth and survival in chemically dynamic environments.

Consistent with this pattern, metal resistance genes were broadly but unevenly distributed across the pangenome (Fig. S3a). In total, 57 metal resistance genes were identified, of which 33 belong to the cloud genome and only 17 are part of the conserved core. A Mantel test revealed a weak correlation between the core phylogenetic tree and the metal resistance gene matrix (*r* = 0.1248, *P* = 0.033), indicating that resistance profiles are shaped largely by non-vertical processes such as HGT and environmental selection rather than strict phylogenetic inheritance.

A key distinction emerges when comparing species-level and strain-level perspectives. While the *R. erythropolis* pangenome depends on the cloud genome to introduce novel resistance traits, individual strains retain a substantial fraction of resistance genes within their core genome. In strain XP, for example, approximately 65% of metal resistance genes are core-encoded (Fig. S3b), suggesting that the core genome provides basal tolerance essential for survival. To determine how this conserved machinery responds under stress *in vivo*, we next experimentally characterized the resistance phenotypes of strain XP.

### Stress tolerance and morphological plasticity of the model strain XP

To connect genomic predictions with physiological performance, we selected *R. erythropolis* strain XP as a representative experimental model. This allowed direct assessment of whether the extensive resistance repertoire inferred from the pangenome confers measurable phenotypic advantages.

**Fig 3:**
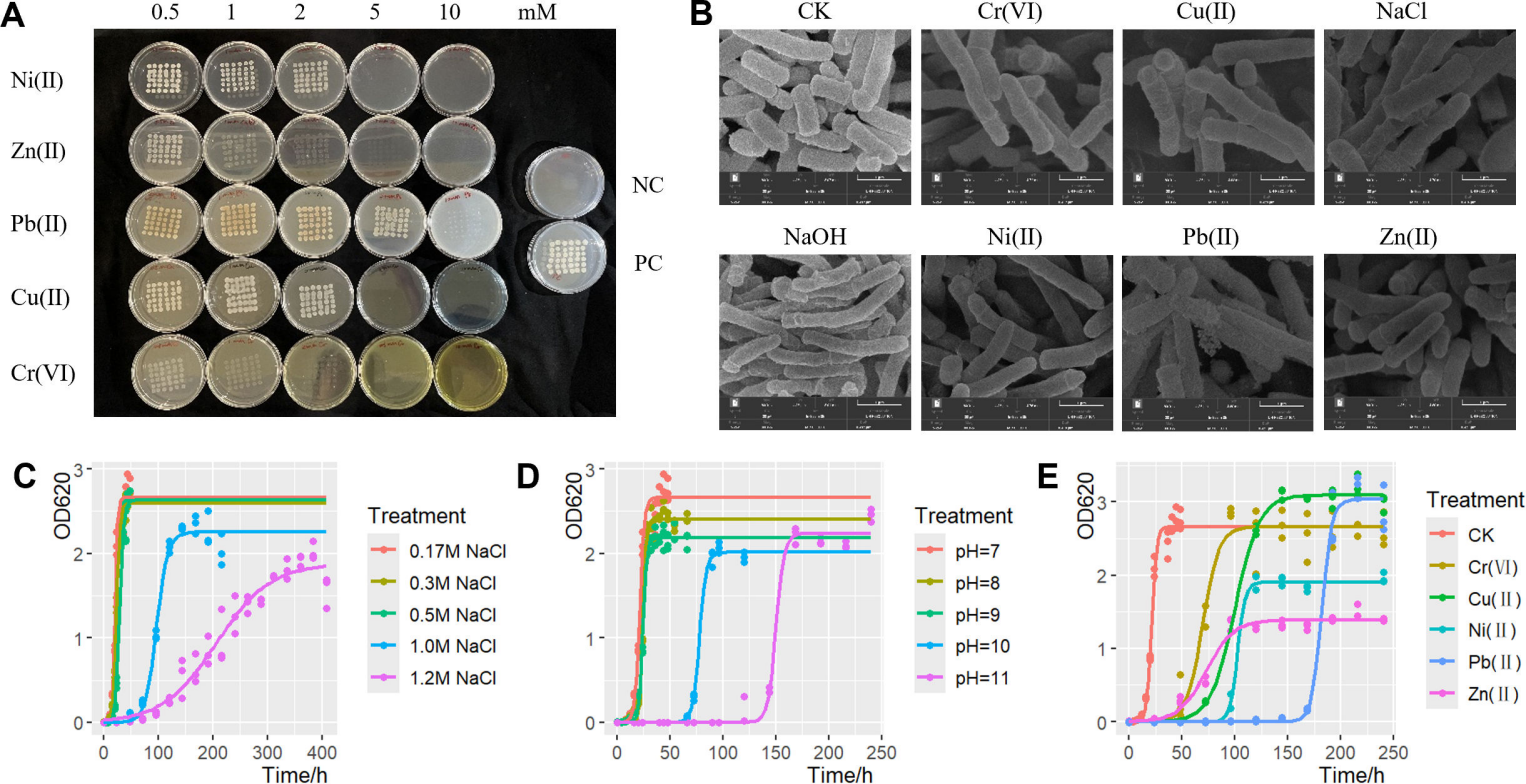
Phenotypic characterization of *R. erythropolis* XP under stress conditions. (**A**) Minimum inhibitory concentrations for Ni(II), Zn(II), Pb(II), Cu(II), and Cr(VI). (**B**) SEM images showing cell morphology under Cr(VI), Cu(II), Ni(II), Pb(II), Zn(II), NaCl, and alkaline (NaOH) stress. (**C–E**) Growth curves under varying NaCl concentrations (**C**), pH levels (**D**), and metal stresses (**E**), fitted to a logistic growth model.

Strain XP displayed exceptional tolerance to multiple heavy metals, with minimum inhibitory concentrations (MICs) of 7 mM for Zn(II) and Pb(II), 4 mM for Cu(II), 3 mM for Ni(II), and 2 mM for Cr(VI) (Fig. 3A; Fig. S4). These values markedly exceed those reported for reference strains; for example, Pb(II) tolerance is approximately 140-fold higher than that of *R. erythropolis* KX814448 (0.05 mM), while Cu(II) and Zn(II) tolerance is increased by four- to sevenfold (30). Beyond metal stress, strain XP grew robustly across a broad pH range (5–11) and tolerated salinity up to 1.5 M NaCl (Fig. 3C and D), confirming the extensive environmental resilience predicted by its open pangenome.

This robustness, however, is accompanied by clear physiological costs. Growth kinetics revealed pronounced trade-offs under stress: although Zn(II) and Pb(II) shared similarly high MICs, they elicited distinct growth dynamics (Fig. 3E). In general, heavy metal exposure prolonged the lag phase and reduced stationary-phase biomass, indicating a diversion of metabolic resources from growth toward activation of resistance mechanisms.

Scanning electron microscopy further revealed striking morphological plasticity in response to environmental stress (Fig. 3B). High salinity induced surface crumpling consistent with osmotic dehydration, whereas exposure to Pb(II), Cu(II), and Cr(VI) triggered pronounced cellular elongation (filamentation) relative to unstressed controls (Fig. S5). Stress-induced filamentation is a hallmark of inhibited cell division, implying that biomass accumulation continues while division machinery is repressed. This phenotype provides morphological evidence for a systemic growth–resistance trade-off and motivated deeper investigation of the underlying transcriptional regulation.

### Global transcriptional reprogramming under environmental stress

**Fig 4:**
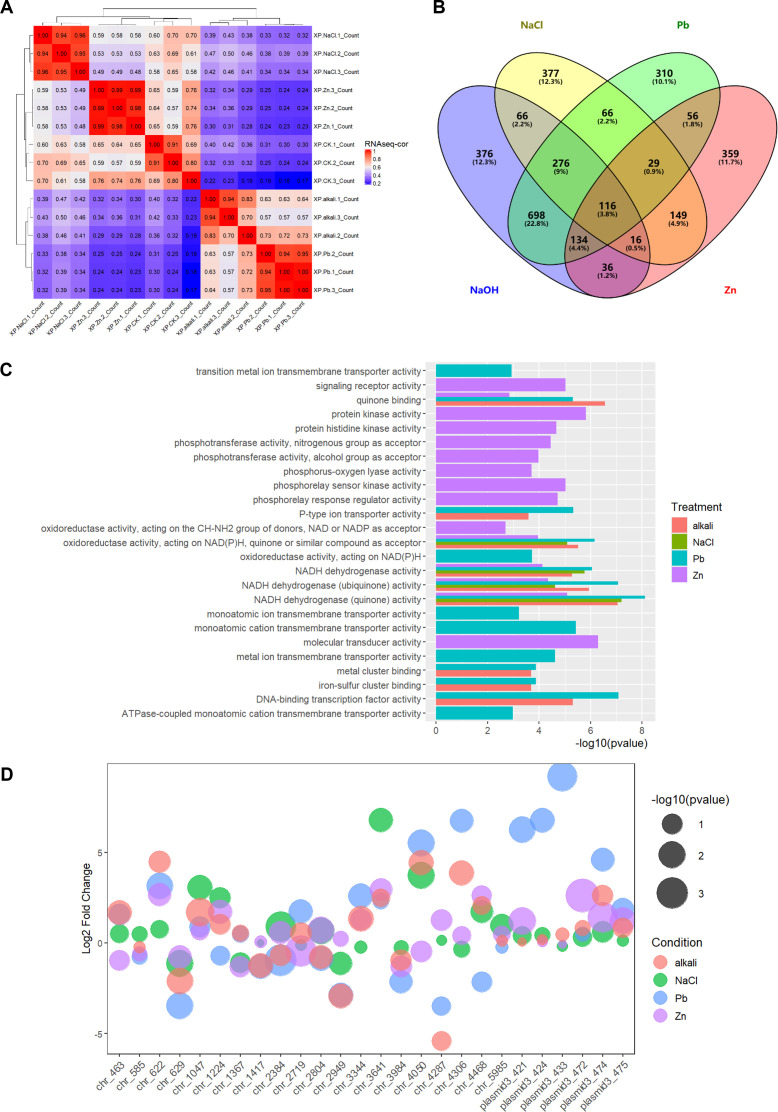
Transcriptomic responses of *R. erythropolis* XP to environmental stress. (**A**) Correlation of RNA-seq biological triplicates under different stress conditions. (**B**) Venn diagram showing the overlap of upregulated genes across conditions. (**C**) GO molecular function enrichment for different stress treatments. (**D**) Expression changes of annotated metal resistance genes across treatment groups.

To dissect the molecular basis of the pronounced physiological robustness of strain XP, we performed RNA-seq under alkaline, high-salinity, Pb(II), and Zn(II) stress conditions. Correlation clustering and principal component analysis revealed that transcriptomes under strong alkaline and Pb(II) stress diverged most strongly from the control, indicating the most extensive global transcriptional reprogramming (Fig. 4A; Fig. S6).

Differential expression analysis quantified the magnitude of these responses. Strong alkaline stress upregulated 1,718 genes (*q* < 0.05, log_2_FC ≥ 1), whereas Pb(II) stress induced 1685 upregulated genes. Zn(II) and high-salinity treatments also elicited substantial responses, with 1,686 and 1,095 genes upregulated, respectively (Fig. 4B). Despite these condition-specific signatures, 116 genes were consistently upregulated across all treatments, defining a shared core stress response.

Functional enrichment of this conserved gene set revealed a common dependence on energy metabolism and redox homeostasis (Fig. 4C). In particular, the uniform induction of NADH dehydrogenase genes points to enhanced ATP generation and intensified NAD^+^/NADH cycling. This suggests that, irrespective of stress type, survival is underpinned by elevating cellular energy capacity to sustain repair processes and maintain oxidative balance.

Superimposed on this core program, each stressor activated distinct adaptive pathways. Under alkaline stress, genes encoding P-type ion transporters and iron–sulfur cluster-binding proteins were preferentially upregulated, consistent with efforts to stabilize proton motive force and metal homeostasis under ionic imbalance. Zn(II) stress was characterized by enrichment of phosphorylation-dependent signaling pathways, implying that zinc toxicity—known to disrupt proteins and induce oxidative stress—is countered through rapid signal transduction to coordinate protective responses. In contrast, Pb(II) stress triggered a detoxification-centric program dominated by cation transporters, indicating that active efflux of lead ions constitutes the primary defense strategy.

To validate these inferences, we examined resistance genes annotated in the MEGARes database (31). In agreement with functional predictions, key metal resistance genes located on plasmids—specifically plasmid3-433, plasmid3-424, and plasmid3-421—were strongly induced, particularly under Pb(II) stress (Fig. 4D). This confirms that the resistance determinants identified *in silico* are transcriptionally mobilized in response to environmental challenge.

### Gene modules linking transcriptional reprogramming to fitness trade-offs

**Fig 5:**
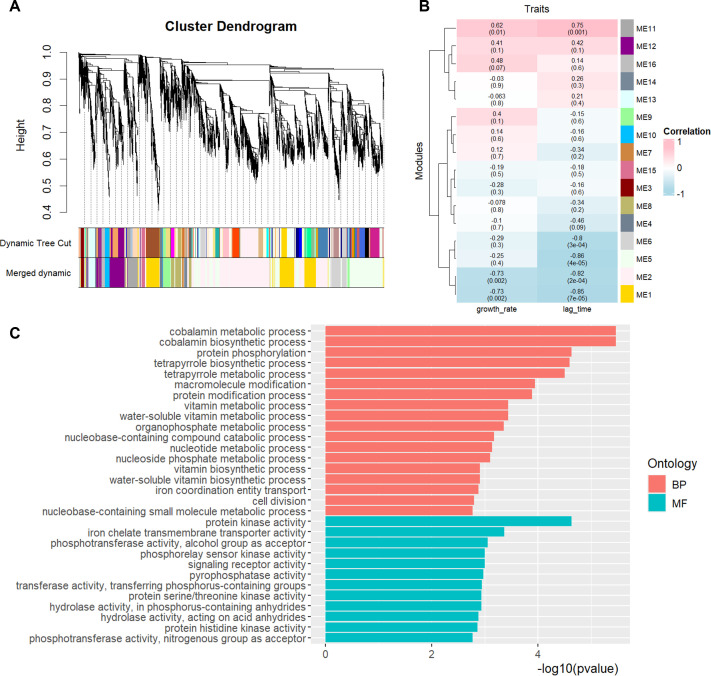
Weighted gene co-expression network analysis of *R. erythropolis*. (**A**) Hierarchical clustering of genes with dynamic tree cut identifying 16 co-expression modules. (**B**) Correlation of each module with growth phenotypes (growth rate and lag time) and associated significance. (**C**) GO enrichment of high-confidence hub genes in module ME1, with statistical significance shown on a logarithmic scale.

The pronounced transcriptional divergence observed under strong alkaline and Pb(II) stress (Fig. 4A) mirrors the substantial physiological costs detected in these conditions, notably prolonged lag phases and reduced growth. To interpret these patterns at a system level, we constructed a WGCNA. This analysis grouped 6,858 genes into 16 co-expression modules, ranging from 40 to 2,071 genes in size (Fig. 5A).

Correlation of module eigengenes with growth traits identified module ME1 as a central determinant of cellular proliferation. ME1 expression was strongly associated with growth kinetics (Fig. 5B) and was markedly suppressed in the slow-growing alkaline and Pb(II) treatments while remaining relatively high under Zn(II) and NaCl stress. Intramodular analysis confirmed the coherence of ME1, revealing a strong positive relationship between module membership (MM) and gene significance (GS) (Fig. S7).

Functional enrichment of high-confidence ME1 hub genes (MM > 0.8, GS > 0.6) clarified the biological basis of this association (Fig. 5C). ME1 is enriched for core cellular processes, including tetrapyrrole and cobalamin (vitamin B12) metabolism, signal transduction, and, most notably, cell division. Repression of this module under alkaline and Pb(II) stress is fully consistent with SEM observations of stress-induced filamentation (Fig. S5), linking transcriptional inhibition of division machinery to the observed elongation phenotype.

In contrast, the Zn(II) and NaCl treatments illustrate a more nuanced relationship between transcription and phenotype. Both conditions maintained high ME1 expression, indicative of an attempt to sustain cell division. However, global correlation analysis showed that while both transcriptomes remain moderately similar to the control, they differ substantially from each other (Fig. 4A). In the Zn(II) condition, this transcriptional configuration supported normal cell morphology, reflecting effective mitigation of zinc toxicity. Under high salinity, however, cells displayed elongation and surface deformation despite elevated ME1 expression, suggesting that while ME1 activity is necessary for normal division, it is insufficient to preserve morphology under osmotic stress, where physical constraints likely override transcriptional control.

To place ME1 in an evolutionary context, we mapped its constituent genes onto pangenome categories. Strikingly, 68.8% of ME1 genes belong to the evolutionarily conserved core and soft-core genomes (584 and 64 genes, respectively), whereas only 20.0% are derived from the cloud genome. This distribution exposes a fundamental evolutionary trade-off: although *R. erythropolis* relies on the variable cloud genome to acquire specialized resistance traits, survival under severe stress is achieved by dynamically downregulating conserved core machinery that drives growth. The repression of ME1 thus represents a calculated cost, sacrificing rapid proliferation to prioritize persistence.

## DISCUSSION

### Taxonomic refinement reveals an open pangenome shaped by environmental adaptation

Genome-wide ANI analysis resolved the taxonomic ambiguity within *Rhodococcus* (26), enabling a high-confidence genomic framework that reveals an open pangenome architecture (32). This open structure reflects a distinct evolutionary strategy: *R. erythropolis* harbors an expansive accessory genome (>70% of genetic diversity), and functional COG analysis (Fig. S8) underscores a clear division of labor—the core genome is enriched in essential housekeeping functions such as translation (category J), whereas the cloud genome is biased toward environmentally responsive functions. Targeted annotation (Fig. S9) further highlights this adaptive capacity: the pangenome encodes dehalogenases, β-glucosidases, and enzymes of aromatic catabolism (catechol and protocatechuate pathways), as well as aerobic CO dehydrogenase, Ni–Fe hydrogenase (group 1), and nitrite reductases, enabling energy acquisition under oligotrophic conditions. Together, these features indicate a highly plastic genome continuously reshaped by HGT, providing the structural basis for the broad physiological adaptability of *R. erythropolis* (33).

### Plasmid-mediated resistance and the accessory gene repertoire

The adaptive potential inferred from pangenome architecture is directly reflected in the resistance mechanisms of strain XP. A dense cluster of heavy metal resistance and redox-related genes on plasmid 3 underscores the central role of extrachromosomal elements as vectors of the accessory genome. This cluster includes *cadA*, which encodes a P-type ATPase for Cd(II)/Pb(II) efflux (34, 35), and *arsAB*, which mediates arsenite extrusion (36). Importantly, these transport systems are co-localized with oxidative stress response genes such as *trxB* (thioredoxin reductase) and *ctpA*, suggesting coordinated evolution to counter both metal toxicity and metal-induced reactive oxygen species (37). Most of these determinants belong to the accessory genome, reinforcing HGT as the dominant mechanism for resistance acquisition and enabling strain XP to persist in multi-metal-contaminated environments.

### Growth-defense trade-offs and resource allocation under stress

Survival in extreme environments depends not only on the presence of resistance genes but also on the strategic redistribution of cellular resources. The growth–defense trade-off theory posits that organisms must balance investment in proliferation against investment in stress tolerance (38–40). Similar patterns have been reported in other bacteria: in *E. coli*, high growth rates are associated with downregulation of stress response genes and upregulation of ribosomal genes (41), and a “fear versus greed” trade-off is characterized by a strong negative correlation between RpoS (stress) and translational (growth) iModulons (42). Under resource limitation, cells divert energy toward repair and adaptation, depleting resources available for proliferation (43). Our multi-omics analyses provide direct evidence that *R. erythropolis* XP adopts this strategy.

Specifically, under severe stress, the bacterium downregulates the growth-associated module ME1 (predominantly core genes like *ftsW* and *prrB*/*prrA*), leading to reduced growth rates and stress-induced filamentation (44). Although *R. erythropolis* iModulons have not yet been characterized, WGCNA-identified ME1 serves as a functional analog. We propose that under lethal or near-lethal stress, the bacterium transiently represses core genome-encoded division and metabolic functions (ME1), conserving energy for expression of accessory resistance determinants such as plasmid-borne *cadA*. This dynamic transcriptional rebalancing—sacrificing vertical propagation to prioritize survival through horizontally acquired defenses—captures the evolutionary logic underlying the exceptional resilience of *R. erythropolis* in extreme environments.

## MATERIALS AND METHODS

### Genome search and filtering

All RefSeq-annotated genome sequences for the genus *Rhodococcus* available in NCBI up to 2024 were retrieved (https://www.ncbi.nlm.nih.gov/). Using NCBI internal filters to exclude atypical assemblies and MAGs, we retained a total of 671 *Rhodococcus* genomes. Pairwise ANI was calculated with fastANI (v1.1) (27). Agglomerative clustering was performed using the average linkage algorithm implemented in the scikit-learn package (AgglomerativeClustering) (45), applying ANI thresholds from 85% to 97% in 0.1% increments to evaluate species boundaries. Clustering patterns and species groupings were visualized using the R package ComplexHeatmap (46).

### Pangenome construction and HGT analysis

Genomes of 52 reclassified *Rhodococcus erythropolis* strains were first assessed for assembly quality using BUSCO (v5.8.2) (47, 48), which estimates completeness, fragmentation, and missing genes. Genomes with ≥95% completeness were retained for pangenomic analysis. After removing incomplete assemblies, genomes with large deletions, and redundant genomes from the same strain, 43 high-quality genomes were selected for final pangenome construction. To minimize annotation bias, all genomes were reannotated uniformly using Prokka (v1.13) (49). Pangenomic clustering of immediate homologs was conducted with Roary (v3.13.0) (50). Core genome-based phylogenetic trees were inferred using FastTree (v2.1.11) (51).

Pangenome openness was evaluated using the R package micropan (v4.3.3) (52) based on Heaps’ law (*n* = *k N*^−α^), with 500 permutations and 100 repeats. The mean α value was used to classify the pangenome as open (α ≤ 1) or closed (α > 1). Pangenome and core genome accumulation curves were derived from the gene presence–absence matrix. Gene gain and loss dynamics were further analyzed using Panstripe, which relates the number of gene exchange events to branch lengths of the core genome phylogeny using a generalized linear model framework implemented in R (28). A Gaussian distribution was applied instead of a compound Poisson (Tweedie) distribution to improve model fitting.

Functional annotation of the final pangenome was performed using eggNOG-mapper (v2.1.12) with the eggNOG (v5.0) database (53, 54). Metabolic profiling was conducted using the METABOLIC-G module in METABOLIC (v4.0) (55). Metal resistance genes were identified by BLAST+ (v2.9.0) (56) searches against the MEGARes (v3.00) database (31), retaining hits with an *e* value of ≤1 × 10^−5^, an alignment length of >200 bp, and the highest score among multiple matches. Putative horizontally transferred genes were identified using HGTector (v2.0) (29) with thresholds of *e* value <1 × 10^−30^, sequence identity >60%, and coverage >80%. The “self” group was defined as the genus *Rhodococcus* (TaxID: 1827), and the “close” group was defined as the family Nocardiaceae (TaxID: 85025).

### MIC determination of heavy metals

MICs of heavy metals for *R. erythropolis* XP were determined following Clinical and Laboratory Standards Institute guideline M07-A9 (57). Strain XP was cultured in LB medium to an OD_600_ of 0.5 and then diluted 100-fold in sterile LB to a final concentration of approximately 10^7^ CFU mL^−1^. Aliquots (2 μL) were inoculated onto LB agar plates supplemented with 0.5, 1, 2, 5, or 10 mM CuSO_4_, ZnSO_4_, NiSO_4_, Pb(CH_3_COO)_2_, or K_2_Cr_2_O_7_; the MIC interval identified in this initial screen was subsequently refined at 1 mM increments. Each concentration was tested with 36 replicates, and plates were incubated at 25°C for 40–48 h.

### Bacterial morphology under stress conditions

Single colonies of *R. erythropolis* XP were activated overnight in kanamycin (Kan)-resistant LB medium (5 ppm Kan) and subsequently cultured under alkaline (pH = 10), high-salinity (1.0 M NaCl), and MIC-level heavy metal stress conditions. Cells were harvested at mid-to-late exponential phase by centrifugation at 4,000 rpm for 5 min at 4°C, washed three times with saline, and fixed with 0.5% glutaraldehyde for 30 min at 4°C. After two additional saline washes, cells were fixed overnight in 2.5% glutaraldehyde at 4°C. Samples were dehydrated sequentially in 50%, 70%, and 100% ethanol, followed by critical point drying using an EM CPD300 system (Leica, Germany). Platinum coating was applied with a Q150T ES Plus high-vacuum coater (Quorum, UK). Cell morphology was examined using a Raman imaging scanning electron microscope (RISE-MAGNA; Tescan, Czech Republic) at 15 kV, and cell dimensions were quantified using ImageJ (58). Statistical significance of cell length differences between stress conditions and control was determined using a two-tailed Student’s *t*-test, with *P* < 0.05 considered significant.

### Growth performance under environmental stress

Single colonies of *R. erythropolis* XP were pre-cultured overnight in LB medium containing 5 ppm kanamycin and used to assess growth under multiple stress conditions. For pH tolerance assays, LB medium was adjusted to pH 4–11 using 10 M NaOH or 5 M HCl. Three independent shaker flasks were prepared for each pH condition and incubated at 25°C with shaking at 200 rpm. At appropriate intervals, cultures were diluted to OD values between 0.3 and 0.8, and 200 μL aliquots were transferred to 96-well plates to measure absorbance at 620 nm using a Spark multi-functional microplate reader.

Salinity tolerance was evaluated by supplementing LB medium with NaCl to final concentrations of 0.17, 0.2, 0.3, 0.5, 1.0, 1.2, and 1.5 M, and growth curves were measured as described above. For heavy metal stress assays, a sterile 250 mM stock solution of each metal salt [CuSO_4_, ZnSO_4_, NiSO_4_, Pb(CH_3_COO)_2_, or K_2_Cr_2_O_7_] was prepared by filtration through a 0.22 μm membrane and diluted to final concentrations of 3 mM Ni(II), 7 mM Zn(II), 7 mM Pb(II), 4 mM Cu(II), and 2 mM Cr(VI) in LB medium prior to inoculation. Growth curves were fitted to a logistic growth model using the R package deSolve (59) to estimate specific growth rates and maximum biomass, and results were visualized with ggplot2 (60).

### RNA sequencing

RNA sequencing was performed under five experimental conditions, each with three biological replicates. Strain XP was cultured in LB medium supplemented with 7 mM ZnSO_4_, 7 mM Pb(CH_3_COO)_2_, strong alkalinity (pH = 10), or high salinity (1.0 M NaCl) until cultures reached an OD_600_ of 0.8. Cells were harvested by centrifugation at 4,000 rpm for 10 min at 4°C in RNase-free tubes; the supernatant was carefully removed; and pellets were immediately snap-frozen in liquid nitrogen for 20 min before storage at −80°C. Total RNA was extracted using a TRIzol Reagent/RNeasy Mini Kit (Qiagen), and 1 μg of total RNA per sample was used for library preparation. Ribosomal RNA was removed using an rRNA Removal Kit, after which the ribosome-depleted RNA was fragmented and reverse-transcribed to construct mRNA libraries. Paired-end sequencing (150 bp × 2) was carried out on an Illumina HiSeq/NovaSeq platform (Illumina, San Diego, CA, USA). All transcriptome sequencing procedures, including RNA extraction, quality assessment, library construction and purification, library quantification, cluster generation, and sequencing, were performed by GENEWIZ (Suzhou). The raw RNA-seq data generated in this study have been deposited in the NCBI BioProject database under accession number PRJNA1396993.

### RNA-seq data processing and computational analysis

Sequencing quality was assessed using FastQC (v0.10.1). High-quality clean reads were aligned to the reference genome using Bowtie2 (v2.2.6), and gene-level read counts were quantified from paired-end data using HTSeq (v0.6.1p1) based on gene length and total mapped read counts (61, 62). Read counts were normalized to fragments per kilobase of transcript per million mapped reads. Sample correlation and hierarchical clustering were visualized with the R package ComplexHeatmap. Differential gene expression analysis was performed using DESeq2 (v1.26.0) from the Bioconductor suite, which models count data using a negative binomial distribution (63). Overlaps among upregulated gene sets were visualized using Venny (v2.1) (64). GO enrichment analysis was conducted with clusterProfiler (v4.10.1) (65), applying a significance threshold of *P* ≤ 0.05.

### Gene network analysis

WGCNA was conducted using the WGCNA package (v1.73) (66). Prior to network construction, sample outliers were identified and removed. Pairwise gene correlations were calculated, and a weighted adjacency matrix was generated using a soft-thresholding power of 9. Based on the resulting topological overlap matrix, hierarchical clustering was performed, and gene modules were identified using the dynamicTreeCut algorithm with parameters minModuleSize = 30 and MEDissThres = 0.15. Modules were represented as branches of the clustering dendrogram and assigned distinct colors. Changes in growth rate and lag time under different stress conditions were used as phenotypic traits to assess module–trait correlations. MM and GS were calculated for each gene, and candidate genes were selected for downstream functional analysis from modules showing high Pearson correlation coefficients (*P* < 0.05), with thresholds of MM > 0.8 and GS > 0.6.

## Supplementary Material

Reviewer comments

## Data Availability

The raw RNA-sequencing data generated in this study are available in the NCBI BioProject database under accession number PRJNA1396993. The genome sequence of *Rhodococcus erythropolis* XP has been deposited under accession number PRJNA1198704. All additional genome sequences used in this study are publicly available from NCBI GenBank, with accession numbers provided in Data set S1. Custom code and scripts used for pangenome analysis, WGCNA, and figure generation have been deposited in Zenodo (DOI: https://doi.org/10.5281/zenodo.18141444)

## SUPPLEMENTAL MATERIAL

Supplemental Figures msystems.00137-26-s0001.docx Fig. S1 to S9.

OPEN PEER REVIEW reviewer-comments.pdf An accounting of the reviewer comments and feedback.

Data set S1 msystems.00137-26-s0002.csv *Rhodococcus* genus genome list.

ASM does not own the copyrights to Supplemental Material that may be linked to, or accessed through, an article. The authors have granted ASM a non-exclusive, world-wide license to publish the Supplemental Material files. Please contact the corresponding author directly for reuse.
