# Supplementary material for: Systemic trade-offs between core and accessory genomes govern stress adaptation in Rhodococcus erythropolis
Source: mSystems. 2026 Jun 9;11(7):e00137-26. doi: 10.1128/msystems.00137-26 (PMC13386940; doi:10.1128/msystems.00137-26)
Supplement: Supplemental Figures — Fig. S1 to S9. [file msystems.00137-26-s0001.docx]

**Supplemental Figures**


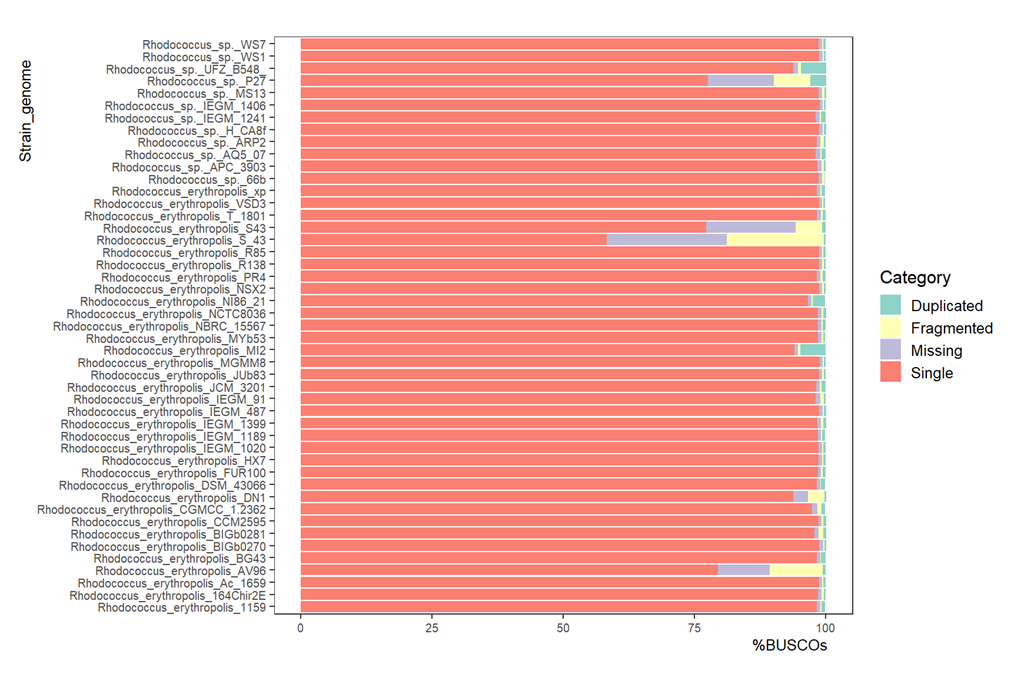


**Fig S1. Genomic integrity assessment.** the percentage of completeness (C), fragmented (F), and missing genes (M) in each genome and genomes with >95% integrity for analysis.


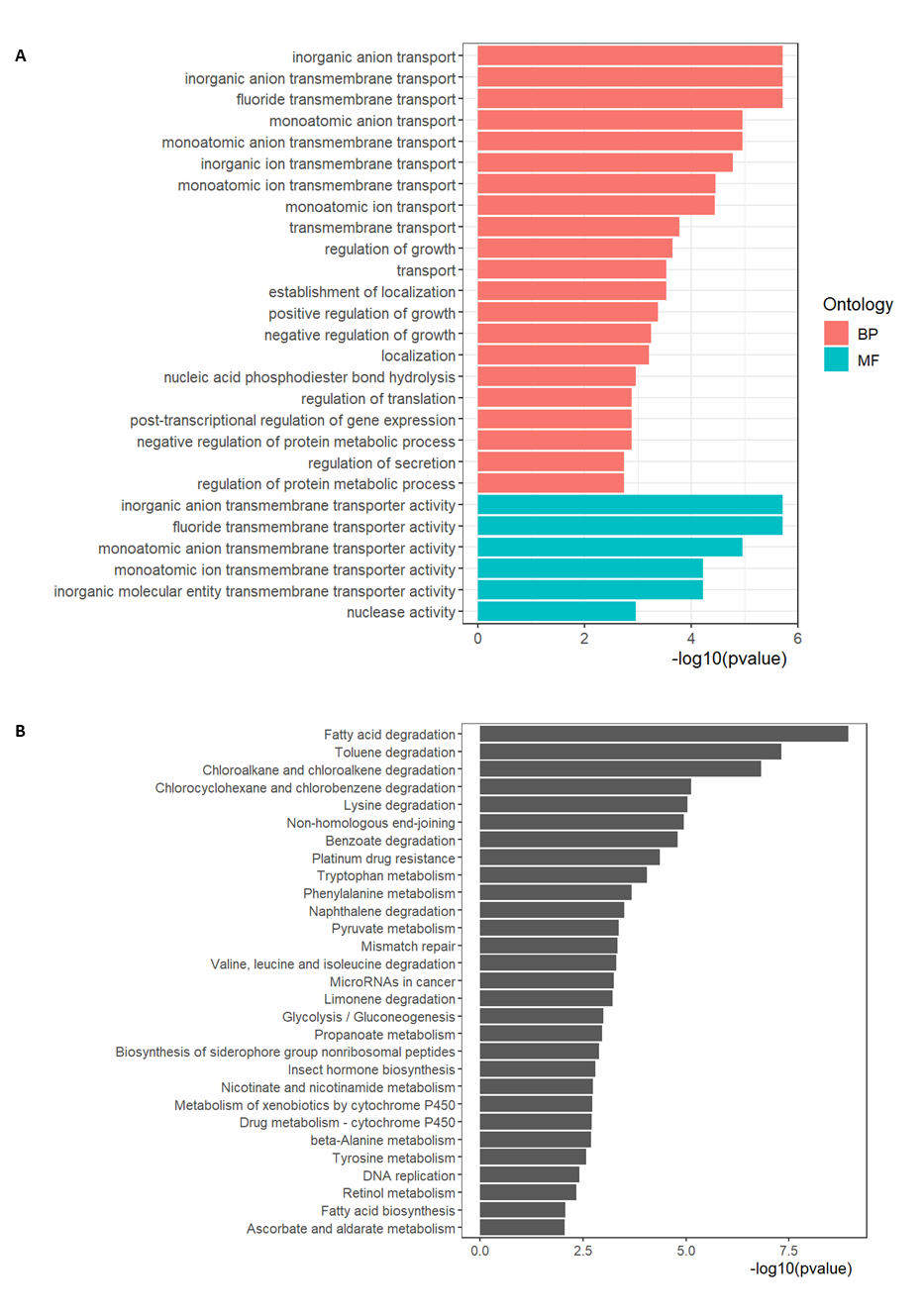


**Fig S2. Gene ontology (GO) and KEGG function enrichment of cloud genes. (A)** GO function of cloud genes with statistical significance (*p* < 0.05). **(B)** KEGG function of cloud genes with statistical significance (*p* < 0.05).


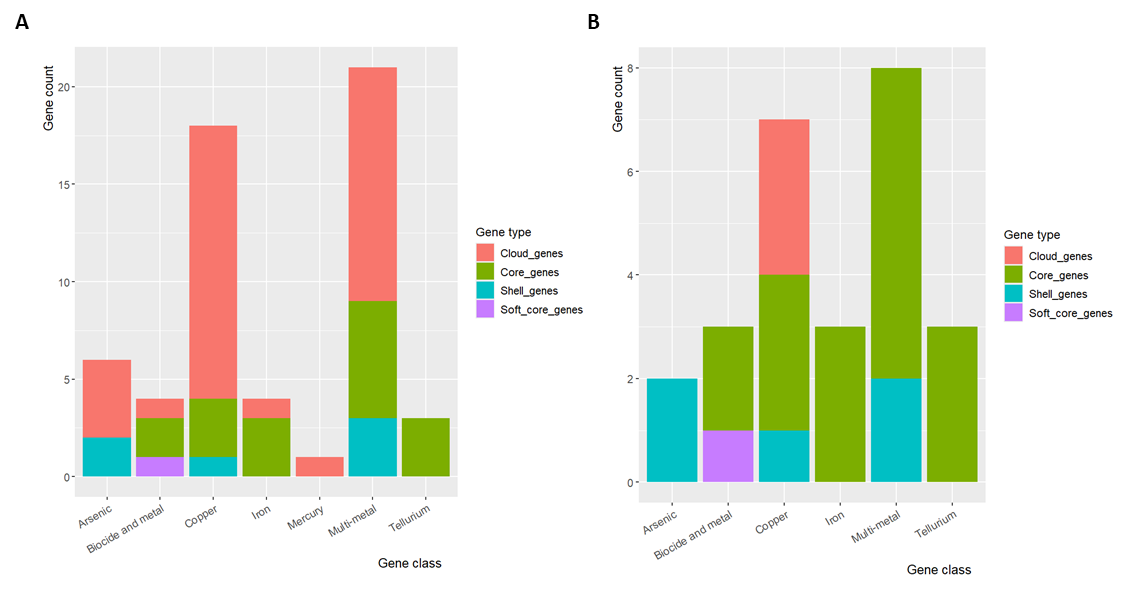


**Fig S3. Distribution of annotated metal resistance genes. (A, B)** Annotated metal resistance genes in *R.erythropolis* pangenome **(A)** and *R.erythropolis* XP **(B).** Red represents cloud genes, green represents core genes, blue represents shell genes and purple represents soft core genes.

**
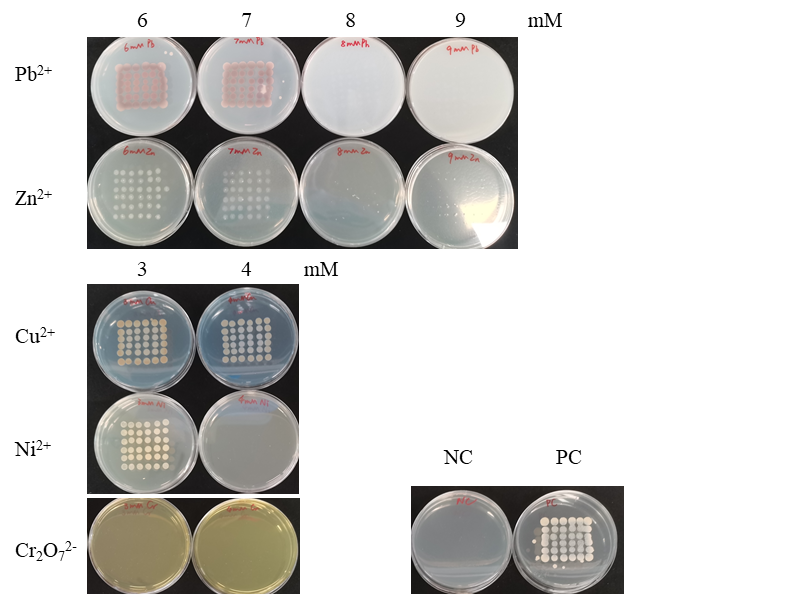
**

**Fig S4. Supplement for minimum inhibitory concentration (MIC) of Ni^2+^, Zn^2+^, Pb^2+^, Cu^2+^ and Cr_2_O_7_^2-^.** Accurate MIC measurement on LB plates with 3-4 or 6-9mm metal added.

**
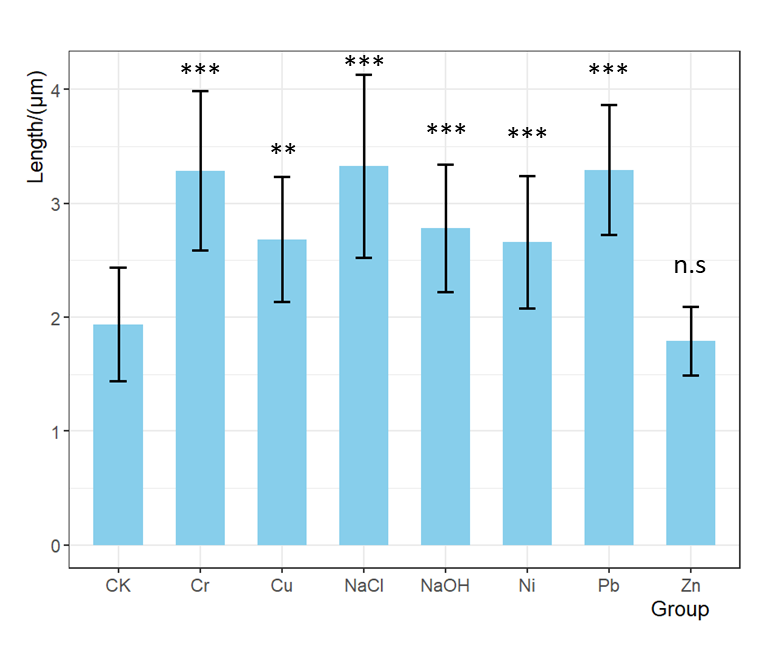
**

**Fig S5. Cell length growing under different stress.** Cell length measured by scanning electron microscopy images. The asterisks represent the significance accoding to obtained *p* value: * *p* < 0.05, ** *p* < 0.01, *** *p* < 0.001.

**
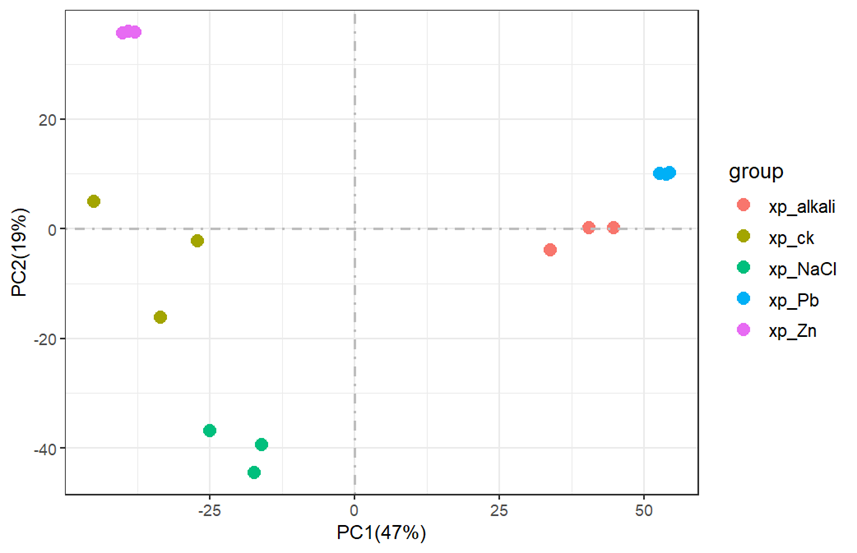
**

**Fig S6. Principal component analysis (PCA) plots.** RNA seq under different stress can be distinguished by PCA plots of biological triplicates growing with PC1 47% and PC2 19%.


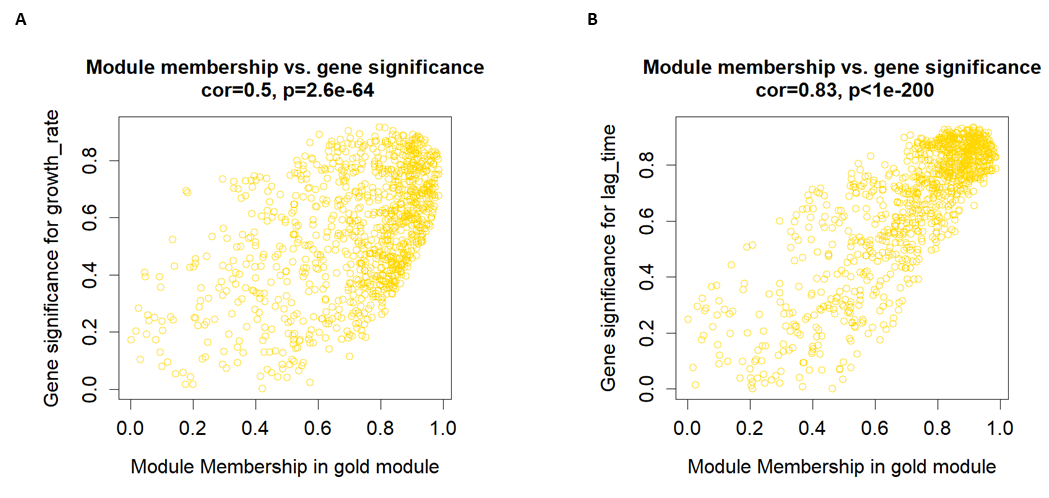


**Fig S7. Corelations between module membership and gene significance in ME1.** The module membership and gene significance (growth rate and lag time) of Genes in ME1 are positively correlated, explaining the relationship between ME1 and these two phenotypes.


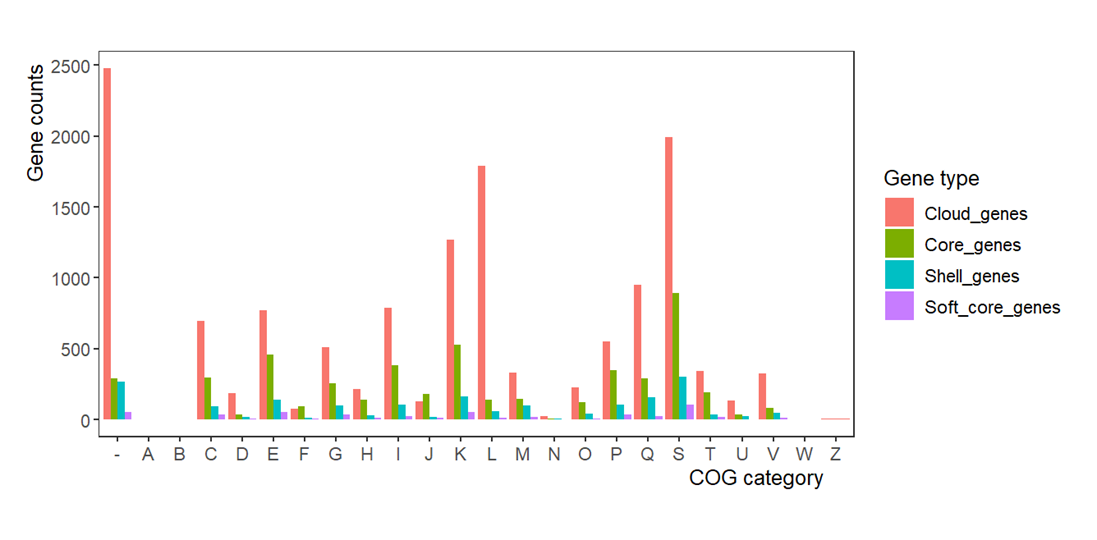


**Fig S8. Distribution of COG function in pangenome based on EggNOG.** Letter code represents the following functional categories: A, RNA processing and modification; B, chromatin structure and dynamics; C, energy production and conversion; D, cell cycle control, cell division, chromosome partitioning; E, amino acid transport and metabolism; F, nucleotide transport and metabolism; G, carbohydrate transport and metabolism; H, coenzyme transport and metabolism; I, lipid transport and metabolism; J, translation, ribosomal structure and biogenesis; K, transcription; L, replication, recombination and repair; M, cell wall/membrane/envelope biogenesis; N, cell motility; O, posttranslational modification, protein turnover, chaperones; P, inorganic ion transport and metabolism; Q, secondary metabolites biosynthesis, transport and catabolism;R, general function prediction only; S, function unknown; T, signal transduction mechanisms; U, intracellular trafficking, secretion and vesicular transport; V, defense mechanisms; W, extracellular structures; X, nan; Y,nuclear structure; Z, cytoskeleton.


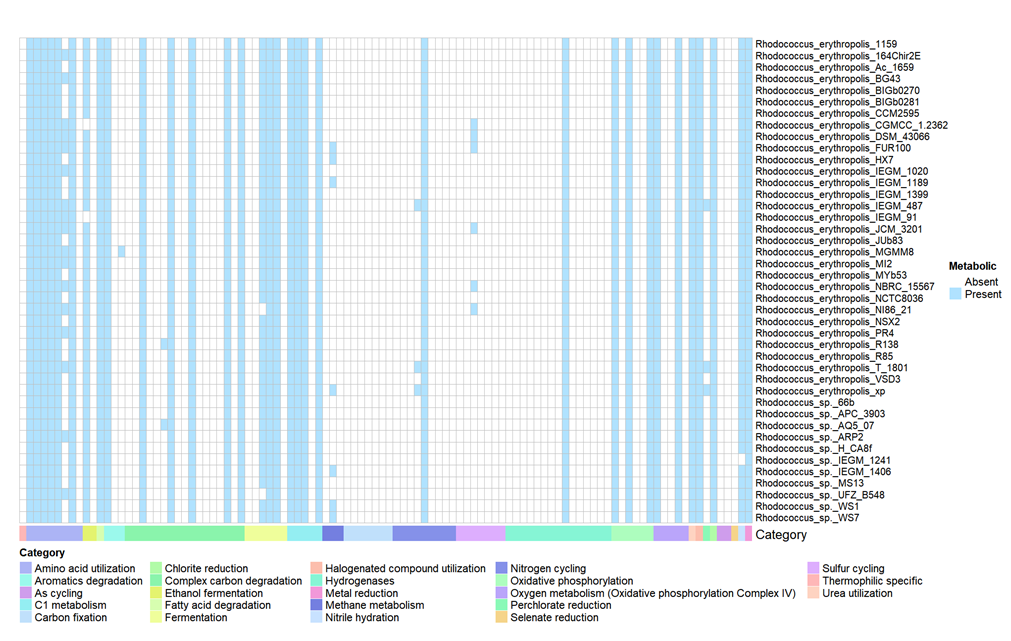


**Fig S9. Metabolic capacity annotation based on METABOLIC.** Blue represents the existence of the function; white represents the nonexistence of the function.
