# Supplementary material for: Systemic trade-offs between core and accessory genomes govern stress adaptation in Rhodococcus erythropolis
Source: mSystems. 2026 Jun 9;11(7):e00137-26. doi: 10.1128/msystems.00137-26 (PMC13386940; doi:10.1128/msystems.00137-26)
Supplement: Reviewer comments [file reviewer-comments.pdf]

AMERICAN SOCIETY FOR MICROBIOLOGY | mSystems®

# Systemic trade-offs between core and accessory genomes govern stress adaptation in *Rhodococcus erythropolis*

Xiaoran Cheng, Huan Liu, Xu Qiu, Wanjing Wu, Haiyang Hu, Ping Xu, and Hongzhi Tang

*Corresponding Author(s): Hongzhi Tang, Shanghai Jiao Tong University*



## Review Timeline:

| | |
|---|---|
| Submission Date: | February 4, 2026 |
| Editorial Decision: | March 11, 2026 |
| Revision Received: | March 24, 2026 |
| Accepted: | April 7, 2026 |

*Editor: Li Cui*

*Reviewer(s): Disclosure of reviewer identity is with reference to reviewer comments included in decision letter(s). The following individuals involved in review of your submission have agreed to reveal their identity: Bo Yu (Reviewer #1); Jian He (Reviewer #2)*

## Transaction Report:

(Note: With the exception of the correction of typographical or spelling errors that could be a source of ambiguity, letters and reports are not edited. The original formatting of letters and referee reports may not be reflected in this compilation.)

**DOI: https://doi.org/10.1128/msystems.00137-26**

Re: mSystems00137-26 (**Systemic trade-offs between core and cloud genomes govern stress adaptation in *Rhodococcus erythropolis***)

Dear Prof. Hongzhi Tang:

Thank you for the privilege of reviewing your work. Below you will find my comments, instructions from the mSystems editorial office, and the reviewer comments.

Please return the manuscript within 60 days; if you cannot complete the modification within this time period, please contact me. If you do not wish to modify the manuscript and prefer to submit it to another journal, notify me immediately so that the manuscript may be formally withdrawn from consideration by mSystems.

**Revision Guidelines**
To submit your modified manuscript, log into the submission site at https://msystems.msubmit.net/cgi-bin/main.plex. Go to Author Tasks and click the appropriate manuscript title to begin. The information you entered when you first submitted the paper will be displayed; update this as necessary. Note the following requirements:

• Upload point-by-point responses to the issues raised by the reviewers in a file named "Response to Reviewers," NOT in your cover letter.
• Upload a compare copy of the manuscript (without figures) as a "Marked-Up Manuscript" file.
• Upload a clean .DOC/.DOCX version of the revised manuscript and remove the previous version.
• Each figure must be uploaded as a separate, editable, high-resolution file (TIFF or EPS preferred), and any multipanel figures must be assembled into one file.
• Any supplemental material intended for posting by ASM should be uploaded with their legends separate from the main manuscript. You can combine all supplemental material into one file (preferred) or split it into a maximum of 10 files with all associated legends included.

For complete guidelines on revision requirements, see our Submission and Review Process webpage. Submission of a paper that does not conform to guidelines may delay acceptance of your manuscript.

**Data availability:** ASM policy requires that data be available to the public upon online posting of the article, so please verify all links to sequence records, if present, and make sure that each number retrieves the full record of the data. If a new accession number is not linked or a link is broken, provide mSystems production staff with the correct URL for the record. If the accession numbers for new data are not publicly accessible before the expected online posting of the article, publication may be delayed; please contact production staff (mSystems@asmusa.org) immediately with the expected release date.

**Publication Fees:** For information on publication fees and which article types are subject to charges, visit our website. If your manuscript is accepted for publication and any fees apply, you will be contacted separately about payment during the production process; please follow the instructions in that e-mail. Arrangements for payment must be made before your article is published.

**ASM Membership:** Corresponding authors may join or renew ASM membership to obtain discounts on publication fees. Need to upgrade your membership level? Please contact Customer Service at Service@asmusa.org.

The ASM Journals program strives for constant improvement in our submission and publication process. Please tell us how we can improve your experience by taking this quick Author Survey.

Thank you for submitting your paper to mSystems.

Sincerely,
Li Cui
Editor
mSystems

Reviewer #1 (Comments for the Author):

This study integrates pangenomics, multi-omics analyses, and phenotypic characterization to reveal the evolutionary mechanisms by which Rhodococcus erythropolis achieves broad environmental tolerance and bioremediation capacity. The results highlight the important role of horizontal gene transfer in driving its environmental adaptability. In addition, the authors use the Rhodococcus erythropolis XP strain as a representative model to further elucidate this environmental tolerance strategy.

Overall, this work represents a typical example of using pangenomic approaches to dissect bacterial physiological phenotypes at a systems level. It reveals an adaptive mechanism to environmental pressures that involves trade-offs between the core genome and the accessory cloud genome. The study is comprehensive and well organized, supported by solid datasets and clear writing, and therefore holds significant scientific and practical value. The following specific comments are provided to help further improve the manuscript.
1. The authors should reconsider and reorganize the logical flow of the Introduction section. In the second paragraph, the introduction to pangenome research lacks sufficient depth, and the question raised at the end of the paragraph is not further clarified or developed. In the third paragraph, the connection to the pangenome discussion in the previous section is missing. Instead, the text shifts abruptly to an introduction of Rhodococcus erythropolis without establishing a clear transition. Overall,the authors should further refine and strengthen the Introduction to improve its logical coherence and to help readers better understand both the significance of this study and the current state of research in the field of pangenomics.
2. 112-116: It should be clarified how the 14 genomes were identified and retrieved.
3. the front size of fig3 panel C D and E is too small.
4. the discussion can be shorter for better reading experience.

Reviewer #2 (Comments for the Author):

This manuscript redefined the taxonomy of R. erythropolis, an important biotechnological chassis, through large-scale genomics, constructed a high-quality pangenome, and revealed the stress tolerance, morphological plasticity and physiological robustness of the model strain R. erythropolis XP. Totally, the manuscript is very interesting and well organized, and provide theoretical basis for the application of R. erythropolis in biotechnology. However, several key aspects require clarification and strengthening before the work can be considered for publication.
major comments：
1. Line 63-70, The authors introduced what can be achieved through pangenomic analysis and how to conduct it. However, readers may also be interested in what has already been accomplished using this approach. Please revise this part accordingly.
2. The discussion and results sections contain overlapping content; consider streamlining these sections.
3. Regarding the "Growth-defense trade-offs and resource allocation under stress" discussion at the end of the section, it would be worth considering whether similar findings have been reported. If so, please incorporate this information accordingly.
minor comments
1. Line 123, "the" total diversity.
2. Fig. 2 panel b, the numbers are overlapped.

## Title Refinement

Please note a minor refinement to the manuscript title: We have updated the title from "Systemic trade-offs between core and cloud genomes govern stress adaptation in *Rhodococcus erythropolis*" to "Systemic trade-offs between core and accessory genomes govern stress adaptation in *Rhodococcus erythropolis*". This adjustment utilizes the more universally recognized pangenomic terminology ("accessory" instead of "cloud") and better represents the full scope of the horizontally acquired stress-resistance determinants discussed in our study.

## Response to Reviewer 1

This study integrates pangenomics, multi-omics analyses, and phenotypic characterization to reveal the evolutionary mechanisms by which *Rhodococcus erythropolis* achieves broad environmental tolerance and bioremediation capacity. The results highlight the important role of horizontal gene transfer in driving its environmental adaptability. In addition, the authors use the *Rhodococcus erythropolis* XP strain as a representative model to further elucidate this environmental tolerance strategy. Overall, this work represents a typical example of using pangenomic approaches to dissect bacterial physiological phenotypes at a systems level. It reveals an adaptive mechanism to environmental pressures that involves trade-offs between the core genome and the accessory cloud genome. The study is comprehensive and well organized, supported by solid datasets and clear writing, and therefore holds significant scientific and practical value. The following specific comments are provided to help further improve the manuscript.

**Response:** We sincerely thank the reviewer for the positive evaluation and for the constructive comments. Your recognition of the core–cloud genome trade-off mechanism is encouraging. We have revised the manuscript accordingly and provide our point-by-point responses below.

**Comment (1):** The authors should reconsider and reorganize the logical flow of the Introduction section. In the second paragraph, the introduction to pangenome research lacks sufficient depth, and the question raised at the end of the paragraph is not further clarified or developed. In the third paragraph, the connection to the pangenome discussion in the previous section is missing. Instead, the text shifts abruptly to an introduction of *Rhodococcus erythropolis* without establishing a clear transition. Overall, the authors should further

refine and strengthen the Introduction to improve its logical coherence and to help readers better understand both the significance of this study and the current state of research in the field of pangenomics.

**Response:** We thank the reviewer for this helpful feedback. We agree that the original Introduction lacked sufficient depth on pangenomic research and needed a clearer transition to our study system. We have reorganized the Introduction (Lines 66–79) as follows:

1. Pangenome research context: In the second paragraph, we expanded the overview by incorporating classic examples from diverse bacteria (e.g., *Escherichia coli*, *Acinetobacter johnsonii*, *Mycobacterium tuberculosis*, and methicillin-resistant *Staphylococcus aureus*). This highlights how the core–accessory division of labor drives environmental adaptation and resistance (Lines 66–72).

"This approach has revealed core–accessory division of labor in diverse bacteria. For instance, the accessory genome of *Escherichia coli* and *Acinetobacter johnsonii* drives environmental adaptation and habitat-specific traits (17, 18), and variable genome elements of pathogens such as *Mycobacterium tuberculosis* and methicillin-resistant *Staphylococcus aureus* underpin virulence and drug resistance (19, 20)."

2. Scientific question: We explicitly stated the overarching question at the end of the second paragraph (Lines 72–75).

"Despite these advances, how pangenome architecture translates into physiological stress responses, particularly the regulatory logic governing resource allocation between growth and defense, remains underexplored."

3. Transition to *R. erythropolis*: We added the transition to connect the pangenomic framework with our study organism as the following (Lines 76–79).

"Within this genus, *Rhodococcus erythropolis* offers an ideal system to bridge this gap. It is a metabolically versatile species with broad environmental adaptability, underpinned by an enzymatic repertoire that enables diverse bioconversions. (7, 21, 22)"

**Comment (2):** 112-116: It should be clarified how the 14 genomes were identified and retrieved.

**Response:** We thank the reviewer for pointing out this lack of clarity. We have added a clear explanation in the revised manuscript to specify the origin of these 14 genomes (Lines 118–123). Specifically, we clarified that they were retrieved from NCBI and originally annotated ambiguously as *Rhodococcus* sp., but were confidently reclassified into our dataset based on our genome-wide ANI clustering.

"ANI-based clustering identified 52 genomes as *R. erythropolis* sensu stricto; of these, 38 were already annotated as *R. erythropolis* in NCBI, whereas 14 were originally annotated as *Rhodococcus* sp. but clustered with *R. erythropolis*. This curated dataset of 52 high-confidence genomes served as the foundation for subsequent pangenome analyses."

**Comment (3):** the front size of fig3 panel C D and E is too small.

**Response:** We thank the reviewer for pointing this out. We have increased the font sizes of the axis labels, tick marks, and legends in panels C, D, and E of Figure 3 to ensure clear readability.

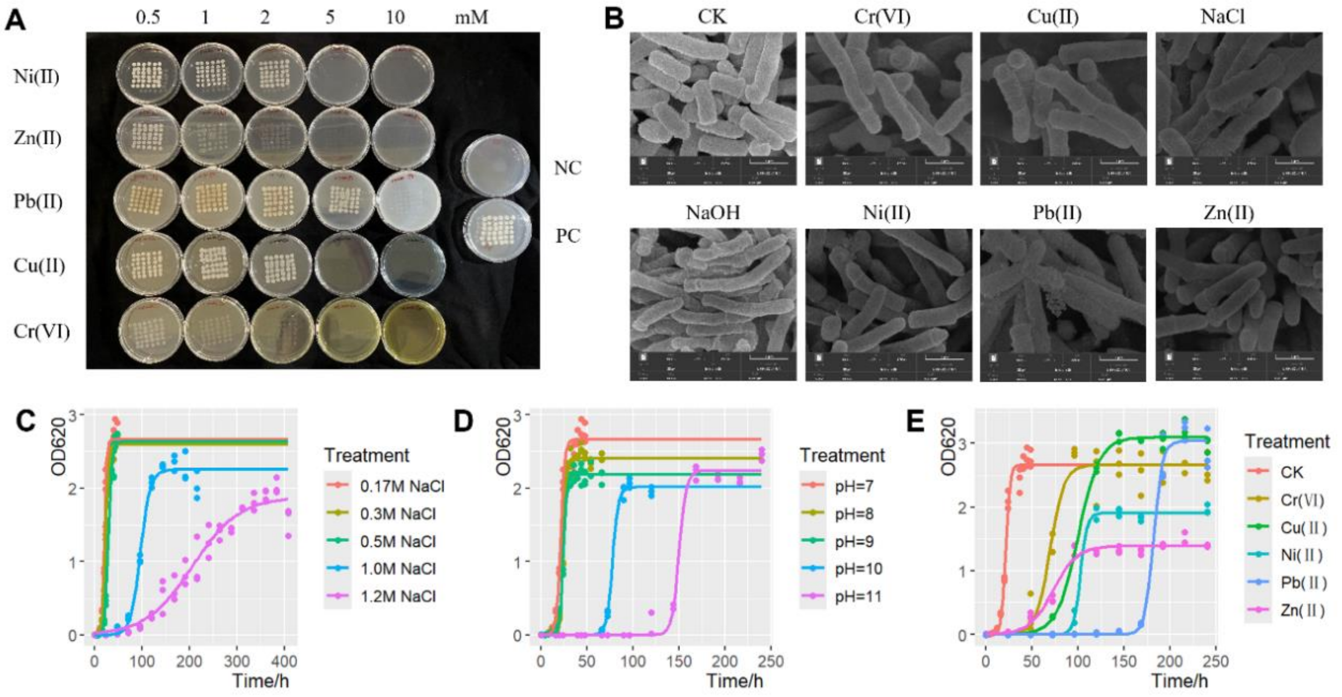


**Fig. 3. Phenotypic characterization of *R. erythropolis* XP under stress conditions. (A)** Minimum inhibitory concentrations (MICs) for Ni(II), Zn(II), Pb(II), Cu(II), and Cr(VI). **(B)** SEM images showing cell morphology under Cr(VI), Cu(II), Ni(II), Pb(II), Zn(II), NaCl, and alkaline (NaOH) stress. **(C–E)** Growth curves under varying NaCl concentrations **(C)**, pH levels **(D)**, and metal stresses **(E)**, fitted to a logistic growth model.

**Comment (4):** the discussion can be shorter for better reading experience.

**Response:** We fully agree with the reviewer that the original Discussion was somewhat lengthy. We have carefully reviewed and streamlined this section to improve the reading experience. Specifically, we removed redundant descriptions of the analytical results and condensed the text across all three subsections:

1. In the first subsection ("Taxonomic refinement reveals an open pangenome shaped by environmental adaptation "): We significantly condensed the text by removing redundant methodological details (e.g., the limitations of 16S rRNA sequencing and the specific steps of excluding misclassified genomes) (Lines 285-300), which are already well-covered in the Introduction and Results section.

"Genome-wide ANI analysis resolved the taxonomic ambiguity within *Rhodococcus* (26), enabling a high-confidence genomic framework that reveals an open pangenome architecture (32). This open structure reflects a distinct evolutionary strategy: *R. erythropolis* harbors an expansive accessory genome (>70% of genetic diversity), and functional COG analysis (Fig. S8) underscores a clear division of labor—the core genome is enriched in essential housekeeping functions such as translation (category J), whereas the cloud genome is biased toward environmentally responsive functions. Targeted annotation (Fig. S9) further highlights this adaptive capacity: the pangenome encodes dehalogenases, β-glucosidases, and enzymes of aromatic catabolism (catechol and protocatechuate pathways), as well as aerobic CO dehydrogenase, Ni–Fe hydrogenase (Group 1), and nitrite reductases, enabling energy acquisition under oligotrophic conditions. Together, these features indicate a highly plastic genome continuously reshaped by horizontal gene transfer (HGT), providing the structural basis for the broad physiological adaptability of *R. erythropolis* (33)."

2. In the second subsection ("Plasmid-mediated resistance and the accessory gene repertoire"): We merged repetitive sentences describing the heavy metal resistance clusters. The revised text now directly highlights the role of extrachromosomal elements and horizontal gene transfer without redundant elaboration (Lines 302-313).

"The adaptive potential inferred from pangenome architecture is directly reflected in the resistance mechanisms of strain XP. A dense cluster of heavy metal resistance and redox-related genes on plasmid 3 underscores the central role of extrachromosomal elements as vectors of the accessory genome. This cluster includes *cadA*, encoding a P-type ATPase for Cd(II)/Pb(II) efflux (34, 35), and *arsAB*, which mediates arsenite extrusion (36). Importantly, these transport systems are co-localized with oxidative stress response

genes such as *trxB* (thioredoxin reductase) and *ctpA*, suggesting coordinated evolution to counter both metal toxicity and metal-induced reactive oxygen species (37). Most of these determinants belong to the accessory genome, reinforcing HGT as the dominant mechanism for resistance acquisition and enabling strain XP to persist in multi-metal–contaminated environments."

3. In the third subsection ("Growth-defense trade-offs and resource allocation under stress"): We streamlined the description of our multi-omics and phenotypic results. Instead of detailing the transcriptomic changes (ME1 module repression), physiological costs (lag phases), and morphological plasticity (filamentation) in separate, lengthy sentences, we integrated these findings into highly concise statements that directly support our conceptual model (Lines 315-337).

"Survival in extreme environments depends not only on the presence of resistance genes but also on the strategic redistribution of cellular resources. The growth–defense trade-off theory posits that organisms must balance investment in proliferation against investment in stress tolerance (38-40). Similar patterns have been reported in other bacteria: in *E. coli*, high growth rates are associated with downregulation of stress response genes and upregulation of ribosomal genes (41), and a "fear versus greed" trade-off is characterized by a strong negative correlation between RpoS (stress) and translational (growth) iModulons (42). Under resource limitation, cells divert energy toward repair and adaptation, depleting resources available for proliferation (43). Our multi-omics analyses provide direct evidence that *R. erythropolis* XP adopts this strategy.

Specifically, under severe stress, the bacterium downregulates the growth-associated module ME1 (predominantly core genes like *ftsW* and *prrB*/*prrA*), leading to reduced growth rates and stress-induced filamentation (44). Although *R. erythropolis* iModulons have not yet been characterized, WGCNA-identified ME1 serves as a functional analog. We propose that under lethal or near-lethal stress, the bacterium transiently represses core genome-encoded division and metabolic functions (ME1), conserving energy for expression of accessory resistance determinants such as plasmid-borne *cadA*. This dynamic transcriptional rebalancing—sacrificing vertical propagation to prioritize survival through horizontally acquired defenses—captures the evolutionary logic underlying the exceptional resilience of *R. erythropolis* in extreme environments."

## Response to Reviewer 2

This manuscript redefined the taxonomy of *R. erythropolis*, an important biotechnological chassis, through large-scale genomics, constructed a high-quality pangenome, and revealed the stress tolerance, morphological plasticity and physiological robustness of the model strain *R. erythropolis* XP. Totally, the manuscript is very interesting and well organized, and provide theoretical basis for the application of *R. erythropolis* in biotechnology. However, several key aspects require clarification and strengthening before the work can be considered for publication.

**Response:** We sincerely thank the reviewer for the highly positive evaluation of our manuscript. We are greatly encouraged by your recognition that our pangenomic and multi-omics analyses provide a solid theoretical basis for the biotechnological application of *R. erythropolis*. We have carefully studied your constructive comments and revised the manuscript to clarify and strengthen the key aspects you pointed out. Please find our point-by-point responses below.

**Comment (1):** Line 63-70, The authors introduced what can be achieved through pangenomic analysis and how to conduct it. However, readers may also be interested in what has already been accomplished using this approach. Please revise this part accordingly.

**Response:**

We thank the reviewer for this constructive suggestion. We completely agree that providing concrete examples of past achievements enhances the background context.

To address this comment, we have expanded the second paragraph of the Introduction to highlight what has already been accomplished using pangenomic approaches. Specifically, we incorporated classic findings from diverse bacteria, such as *Escherichia coli*, *Acinetobacter johnsonii*, *Mycobacterium tuberculosis*, and methicillin-resistant *Staphylococcus aureus* (Lines 66–72). These additions illustrate how previous pangenomic studies have successfully revealed the core–accessory division of labor that drives environmental adaptation and stress/antibiotic resistance. We believe these revisions effectively address your concern and provide readers with a clearer picture of the current achievements in the field of pangenomics.

"This approach has revealed core–accessory division of labor in diverse bacteria. For instance, the accessory genome of *Escherichia coli* and *Acinetobacter johnsonii* drives environmental adaptation and habitat-specific traits (17, 18), and variable genome elements of pathogens such as *Mycobacterium tuberculosis* and methicillin-resistant *Staphylococcus aureus* underpin virulence and drug resistance (19, 20)."

**Comment (2):** The discussion and results sections contain overlapping content; consider streamlining these sections.

**Response:**

We fully agree with the reviewer's observation. To address this issue, we have carefully reviewed and streamlined the Discussion section to eliminate redundant overlaps with the Results. Specifically, we implemented the following changes to ensure the Discussion focuses strictly on biological interpretation rather than restating data:

1. Removed analytical repetitions: In the first subsection, we deleted detailed descriptions of the taxonomic reclassification steps and the exclusion of misclassified genomes, as these are already comprehensively covered in the Results (Lines 285-300).

"Genome-wide ANI analysis resolved the taxonomic ambiguity within *Rhodococcus* (26), enabling a high-confidence genomic framework that reveals an open pangenome architecture (32). This open structure reflects a distinct evolutionary strategy: *R. erythropolis* harbors an expansive accessory genome (>70% of genetic diversity), and functional COG analysis (Fig. S8) underscores a clear division of labor—the core genome is enriched in essential housekeeping functions such as translation (category J), whereas the cloud genome is biased toward environmentally responsive functions. Targeted annotation (Fig. S9) further highlights this adaptive capacity: the pangenome encodes dehalogenases, β-glucosidases, and enzymes of aromatic catabolism (catechol and protocatechuate pathways), as well as aerobic CO dehydrogenase, Ni–Fe hydrogenase (Group 1), and nitrite reductases, enabling energy acquisition under oligotrophic conditions. Together, these features indicate a highly plastic genome continuously reshaped by horizontal gene transfer (HGT), providing the structural basis for the broad physiological adaptability of *R. erythropolis* (33)."

2. Condensed data descriptions: We significantly shortened the repetitive elaboration on specific heavy

metal resistance gene clusters in the second subsection (Lines 302-313).

"The adaptive potential inferred from pangenome architecture is directly reflected in the resistance mechanisms of strain XP. A dense cluster of heavy metal resistance and redox-related genes on plasmid 3 underscores the central role of extrachromosomal elements as vectors of the accessory genome. This cluster includes *cadA*, encoding a P-type ATPase for Cd(II)/Pb(II) efflux (34, 35), and *arsAB*, which mediates arsenite extrusion (36). Importantly, these transport systems are co-localized with oxidative stress response genes such as *trxB* (thioredoxin reductase) and *ctpA*, suggesting coordinated evolution to counter both metal toxicity and metal-induced reactive oxygen species (37). Most of these determinants belong to the accessory genome, reinforcing HGT as the dominant mechanism for resistance acquisition and enabling strain XP to persist in multi-metal–contaminated environments."

3. Synthesized multi-omics findings: In the final subsection, instead of restating the individual transcriptomic changes (ME1 downregulation) and physiological phenotypes (filamentation and lag phases) point-by-point, we merged and condensed them directly into our conceptual model of the "growth-defense trade-off" (Lines 315-337).

"Survival in extreme environments depends not only on the presence of resistance genes but also on the strategic redistribution of cellular resources. The growth–defense trade-off theory posits that organisms must balance investment in proliferation against investment in stress tolerance (38-40). Similar patterns have been reported in other bacteria: in *E. coli*, high growth rates are associated with downregulation of stress response genes and upregulation of ribosomal genes (41), and a "fear versus greed" trade-off is characterized by a strong negative correlation between RpoS (stress) and translational (growth) iModulons (42). Under resource limitation, cells divert energy toward repair and adaptation, depleting resources available for proliferation (43). Our multi-omics analyses provide direct evidence that *R. erythropolis* XP adopts this strategy.

Specifically, under severe stress, the bacterium downregulates the growth-associated module ME1 (predominantly core genes like *ftsW* and *prrB*/*prrA*), leading to reduced growth rates and stress-induced filamentation (44). Although *R. erythropolis* iModulons have not yet been characterized, WGCNA-identified ME1 serves as a functional analog. We propose that under lethal or near-lethal stress, the bacterium transiently represses core genome-encoded division and metabolic functions (ME1), conserving energy for expression of accessory resistance determinants such as plasmid-borne *cadA*. This dynamic

transcriptional rebalancing—sacrificing vertical propagation to prioritize survival through horizontally acquired defenses—captures the evolutionary logic underlying the exceptional resilience of *R. erythropolis* in extreme environments."

These revisions have substantially reduced redundancy and significantly improved the logical flow and readability of the manuscript.

**Comment (3):** Regarding the "Growth-defense trade-offs and resource allocation under stress" discussion at the end of the section, it would be worth considering whether similar findings have been reported. If so, please incorporate this information accordingly.

**Response:**

We thank the reviewer for this insightful suggestion. We completely agree that incorporating similar findings from other organisms significantly strengthens the theoretical foundation and universal relevance of our proposed model.

Following your advice, we have expanded the first paragraph of the "Growth-defense trade-offs and resource allocation under stress" subsection to include classic growth-defense trade-off phenomena reported in other bacteria. Specifically, we incorporated the well-documented "fear versus greed" resource allocation observed in *Escherichia coli* (Lines 318-325), which mirrors the physiological logic we identified in *R. erythropolis*. We believe this addition addresses your comment and embeds our specific findings within a broader, universally recognized biological framework.

"Similar patterns have been reported in other bacteria: in *E. coli*, high growth rates are associated with downregulation of stress response genes and upregulation of ribosomal genes (41), and a "fear versus greed" trade-off is characterized by a strong negative correlation between RpoS (stress) and translational (growth) iModulons (42). Under resource limitation, cells divert energy toward repair and adaptation, depleting resources available for proliferation (43)."

**Minor comment (1):** Line 123, "the" total diversity.

**Response:**

We thank the reviewer for their careful reading and for catching this omission. We have corrected the typographical error by adding "the" before "total diversity" in the revised manuscript (Line 130).

"This analysis identified 23,081 gene families (Fig. 2A), revealing a pangenome overwhelmingly dominated by the accessory cloud genome, which accounts for 70.6% (16,302 families) of the total diversity."

**Minor comment (2):** Fig. 2 panel b, the numbers are overlapped.

**Response:**

We apologize for this visual oversight and thank the reviewer for pointing it out. We have adjusted the layout and spacing in Figure 2 panel b to ensure all numbers are clearly legible without any overlapping. The corrected, high-resolution figure has been provided in the revised submission.

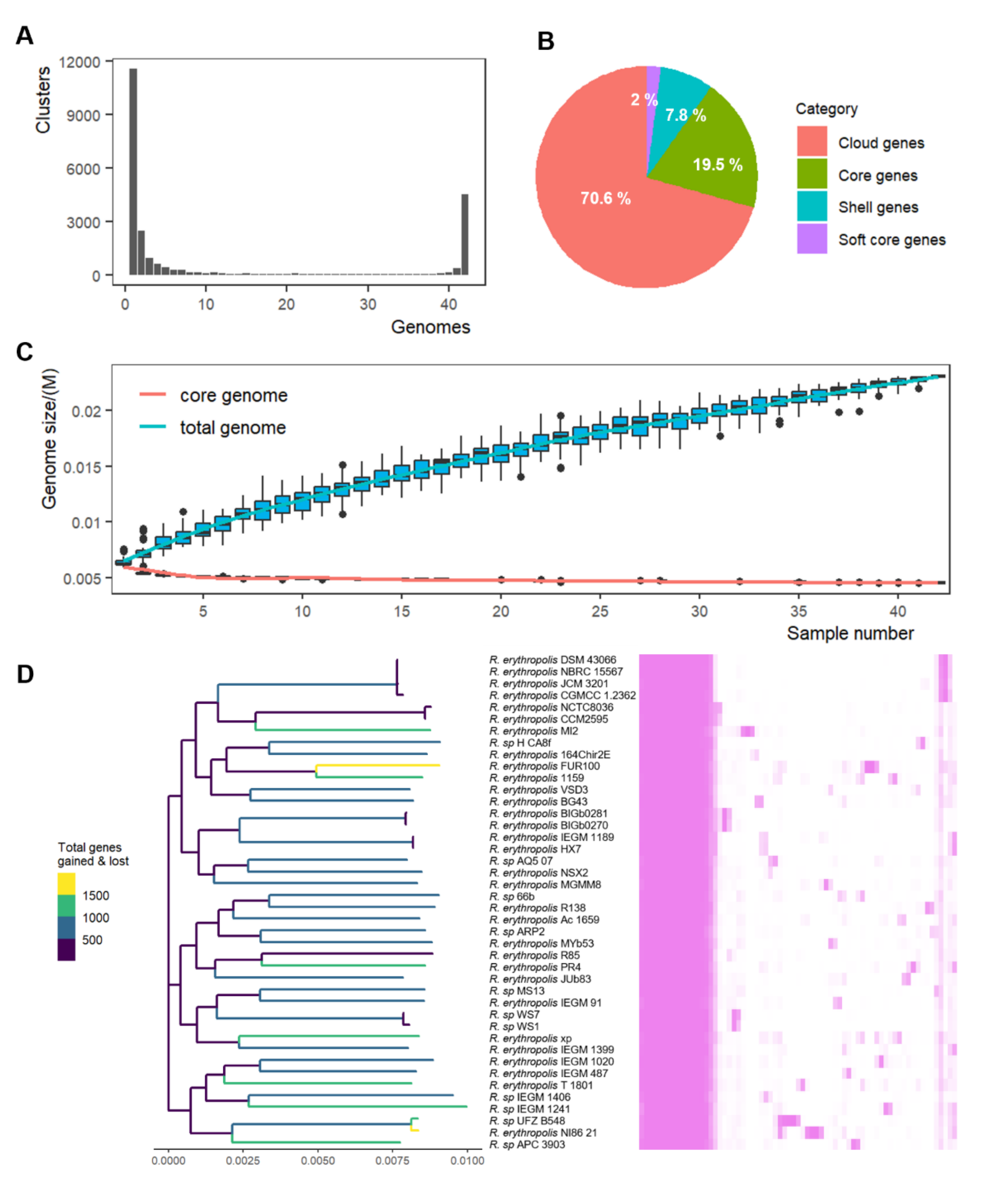


**Fig. 2. Pangenome dynamics of *R. erythropolis*. (A)** Distribution of 23,081 gene clusters across the genomes, showing 11,571 strain-specific clusters and 4,507 core clusters shared by all genomes. **(B)** Proportions of core (n ≥ 99%), soft-core (99% > n ≥ 95%), shell (95% > n ≥ 15%), and cloud (n < 15%) gene clusters. **(C)** Gene accumulation curves for the pangenome (blue) and core genome (red) with sequential genome additions. **(D)** Phylogenetic tree of *R. erythropolis* with branches colored by inferred gene gain and loss events using maximum parsimony. (left) Gene presence/absence matrix across genomes (pink, present; white, absent). (right)

Re: mSystems00137-26R1 (**Systemic trade-offs between core andaccessory genomes govern stress adaptation in *Rhodococcus erythropolis***)

Dear Prof. Hongzhi Tang:

Your manuscript has been accepted, and I am forwarding it to the ASM production staff for publication. Your paper will first be checked to make sure all elements meet the technical requirements. ASM staff will contact you if anything needs to be revised before copyediting and production can begin. Otherwise, you will be notified when your proofs are ready to be viewed.

**Data Availability:** ASM policy requires that data be available to the public upon online posting of the article, so please verify all links to sequence records, if present, and make sure that each number retrieves the full record of the data. If a new accession number is not linked or a link is broken, provide production staff with the correct URL for the record. If the accession numbers for new data are not publicly accessible before the expected online posting of the article, publication may be delayed; please contact ASM production staff immediately with the expected release date.

**Publication Fees:** For information on publication fees and which article types have charges, please visit ourwebsite. We have partnered with Copyright Clearance Center (CCC) to collect author charges. If fees apply to your paper, you will receive a message from no-reply@copyright.com with further instructions. For questions related to paying charges through RightsLink, please contact CCC at ASM_Support@copyright.com or toll free at +1-877-622-5543. CCC makes every attempt to respond to all emails within 24 hours.

**ASM Membership:** Corresponding authors may join or renew ASM membership to obtain discounts on publication fees. Need to upgrade your membership level? Please contact Customer Service at Service@asmusa.org.

**Embargo Policy:** A press release may be issued as soon as the manuscript is posted on themSystems Latest Articles webpage. The corresponding author will receive an email with the subject line "ASM Journals Author Services Notification" when the article is available online.

**Cover Image Submissions:** If you would like to submit a potential Cover Image, please email a file and a short legend to msystems@asmusa.org. Please note that we can only consider images that (i) the authors created or own and (ii) have not been previously published. By submitting, you agree that the image can be used under the same terms as the published article. Image File requirements: TIF/EPS, 7.5 inches wide by 8.25 inches tall (at least 2,250 pixels wide by 2,475 pixels tall), minimum 300 dpi resolution (600 dpi preferred), RGB, and no figure elements, e.g., arrows or panel labels. The legend should be a short description of the image, 1-2 sentences recommended. Please download and use this interactive template in Adobe to ensure that your proposed cover image meets our size requirements (https://journals.asm.org/pb-assets/pdf-text-excel-files/ASM-Interactive-Sizing-Cover-Template-1715689791.pdf).

Thank you for submitting your paper to mSystems.

Sincerely,
Li Cui
Editor
mSystems

Reviewer #1 (Comments for the Author):

The authors well revised the manuscript. No further comments.

Reviewer #2 (Comments for the Author):

The manuscript has been well revised,all questions raised by reviewers have been solved.
